# Discussing Weight Management With Type 2 Diabetes Patients in Primary Care Using the Small Talk Big Difference Intervention: Protocol for a Randomized Controlled Trial

**DOI:** 10.2196/12162

**Published:** 2019-02-15

**Authors:** Katriona Brooksbank, Joanne O'Donnell, Vicky Corbett, Sarah Shield, Rachel Ainsworth, Ross Shearer, Susan Montgomery, Andrew Gallagher, Hannah Duncan, Lorna Hamilton, Valerie Laszlo, Rhonda Noone, Anna Baxendale, David Blane, Jennifer Logue

**Affiliations:** 1 Institute of Cardiovascular and Medical Sciences University of Glasgow Glasgow United Kingdom; 2 Merck Sharpe & Dohme Hoddesdon United Kingdom; 3 AstraZeneca Cambridge United Kingdom; 4 Glasgow and Clyde Weight Management Service Glasgow United Kingdom; 5 National Health Service Greater Glasgow and Clyde Glasgow United Kingdom; 6 Institute of Health and Wellbeing University of Glasgow Glasgow United Kingdom

**Keywords:** obesity, primary care, medical education

## Abstract

**Background:**

Guidelines for the management of type 2 diabetes universally recommend that adults with type 2 diabetes and obesity be offered individualized interventions to encourage weight loss. Yet despite the existing recommendations, provision of weight management services is currently patchy around the United Kingdom and where services are available, high attrition rates are often reported. In addition, individuals often fail to take up services, that is, after discussion with a general practitioner or practice nurse, individuals are referred to the service but do not attend for an appointment. Qualitative research has identified that the initial discussion raising the issue of weight, motivating the patient, and referring to services is crucial to a successful outcome from weight management.

**Objective:**

Our aim was to evaluate the effectiveness of an Internet-based training program and practice implementation toolkit with or without face-to-face training for primary care staff. The primary outcome is the change in referral rate of patients with type 2 diabetes to National Health Service adult weight management programs, 3 months pre- and postintervention.

**Methods:**

We used the Behavior Change Wheel to develop an intervention for staff in primary care consisting of a 1-hour Internet-based eLearning package covering the links between obesity, type 2 diabetes, and the benefits of weight management, the treatment of diabetes in patients with obesity, specific training in raising the issue of weight, local services and referral pathways, overview of weight management components/ evidence base, and the role of the referrer. The package also includes a patient pamphlet, a discussion tool, a practice implementation checklist, and an optional 2.5-hour face-to-face training session. We have randomly assigned 100 practices in a 1:1 ratio to either have immediate access to all the resources or have access delayed for 4 months. An intention-to-treat statistical analysis will be performed.

**Results:**

Recruitment to the study is now complete. We will finalize follow-up in 2018 and publish in early 2019.

**Conclusions:**

This protocol describes the development and randomized evaluation of the effectiveness of an intervention to improve referral and uptake rates of weight management programs for adults with type 2 diabetes. At a time when many new dietary and pharmacological weight management interventions are showing large clinical benefits for people with type 2 diabetes, it is vital that primary care practitioners are willing, skilled, and able to discuss weight and make appropriate referrals to services.

**Trial Registration:**

ClinicalTrials.gov NCT03360058; https://clinicaltrials.gov/ct2/show/NCT03360058 (Archived by WebCite at http://www.webcitation.org/74HI8ULfn)

**International Registered Report Identifier (IRRID):**

DERR1-10.2196/12162

## Introduction

Scottish Intercollegiate Guideline Network guidance recommends that “obese adults with type 2 diabetes should be offered individualized interventions to encourage weight loss (including lifestyle, pharmacological or surgical interventions) in order to improve metabolic control” [1]. Despite the existing recommendations, provision of weight management services is currently patchy around the United Kingdom [2,3]. Where services are available, high attrition rates are often reported [4]. In addition, individuals often fail to take up services even after having seemingly agreed to do so. That is, after discussion with a general practitioner (GP) or practice nurse, individuals are referred to the service but do not attend for an appointment [5].

In the National Health Service (NHS) Greater Glasgow and Clyde Health Board area of Scotland, there are currently 25,109 patients with type 2 diabetes and a body mass index (BMI) ≥30 kg/m^2^, yet only 5855 patients with type 2 diabetes were referred to Glasgow and Clyde Weight Management Service from 2005-2014. Of those, only 1537 attended at the assessment session and only 336 completed the program and lost at least 5 kg [4].

The Glasgow and Clyde Weight Management Service delivers a specialist multidisciplinary, multicomponent, weight management program throughout the Glasgow and Clyde area of the United Kingdom. In an evaluation of the service, the authors highlighted that 27% of the patients who are referred to the program do not opt into the service [6]. This describes patients who are referred via their GP practice and do not contact the service to opt into an initial assessment. Similarly, Brook et al [7] described initial uptake and engagement of a small weight management program of 502 patients. In addition to completing an extensive questionnaire, patients were requested to call to make an appointment with the service personally**.** Of those referred to the program, 46% did not opt in. Engaging patients in a weight management program is especially difficult, even when the intervention is provided via the primary care route. For example, the Counterweight Project, a weight management program delivered via a GP practice, has been taken up by several practices in Scotland. However, after 2 years, one fifth of enlisted practices failed to enroll patients into the program [8].

An explorative focus group study concentrated on patients’ experience of GP management of their weight problems and highlighted how patients would prefer the GP to broach the subject of weight management [9]. Patients were keen to have weight management discussed even when it made them feel embarrassed and they appeared reluctant. Participants highlighted a lack of engagement from GPs regarding weight in addition to poor knowledge regarding service resources for obesity treatment. The authors highlighted the need for GPs to acknowledge the efforts required for long-term lifestyle change while shifting attention from shame to coping. Again, obesity stigma was reported, and the authors highlighted that vulnerable feelings of failure could easily be reinforced by well-intentioned advice. Judgmental attitudes were considered to be particularly demeaning when they came from doctors. In fact, in a recent study of public perceptions of weight-related language used by health providers, 19% of participants highlighted that they would avoid further contact and 21% would seek a new doctor if they felt stigmatized about their weight from their doctor [10].

In fact, GPs may avoid discussion of weight management and lifestyle change altogether as they may feel that they have not received appropriate training to provide effective counseling [11], or they do not approach the subject if patients appear ambivalent about behavior change [12]. Studies also highlight the presence of system-level barriers such as a lack of time during consultations [13]. Even when GPs do address matters of weight-related behavior, there is often disagreement from the patient that the topic has been raised. In a sample of 456 patients, 39% of patients disagreed with GPs’ reporting about the content of the discussion during consultations regarding weight, diet, and physical activity. In particular, GPs reported more occasions of discussing weight than patients in 12.5% of consultations [14]. Patients’ likeliness to engage in a weight management program is also influenced by practice endorsement and opinion of the GP of the intervention available in addition to other factors: clear understanding of the program, clear understanding of the program goals, structured proactive follow-up, and perception of positive outcomes [14].

Given the importance of weight management for type 2 diabetes, we sought to develop and evaluate an intervention to improve referral rates and uptake of weight management programs for patients with type 2 diabetes and co-existent obesity (Textbox 1).

**PICOS summary.**

Participants

Primary care practices in National Health Service Greater Glasgow and Clyde, United Kingdom (at least 1 clinician per practice)

Intervention

A 1-hour eLearning program covering the benefits of weight management in type 2 diabetes, communication skills for raising the issue of weight with patients, and safe management of diabetes during weight loss; patient information pamphlets; patients discussion aid; an implementation toolkit

Comparator

Primary care practices that did not get access to the intervention

Outcomes

Primary – the ratio of referrals over 3 months before and after the Small Talk Big Difference intervention (allowing 1 month for completion)

Secondary – change in referral: uptake ratio; local enhanced service template completion (weight management discussed); change in local enhanced service template completion; completion of lifestyle weight management phase (completion defined as 80% attendance); weight change (kg and %) in lifestyle weight management phase for those attending >1 session; weight change (kg and %) at 1 year for all patients (data from annual diabetes review)

Tertiary – diabetes medications at time of referral: % on weight gaining medications (sulphonylureas, thiazolidinediones, insulin); % on weight neutral/reducing medications (GLP-1 agonists, metformin, DPP-IV inhibitors, SGLT2 inhibitors)

Exploratory – the effect of completion of training; change in referral rate analysis of those who completed Internet-based training only; when training done was completed by GP only, by practice nurse only or both; change in referral rate in those where one practice member completed Internet-based plus face-to-face training

Study Design

Individually randomized controlled trial

## Methods

### Intervention Development

The Behavior Change Wheel [15] was used as a framework to guide the development of the intervention. It provides a systematic approach to better understand the behaviors and effectively target them. It describes 8 separate steps from defining the problem through to mode of delivery [16].

#### Step 1: Define the Problem in Behavioral Terms

Low numbers of patients with type 2 diabetes are currently being referred to weight management [4]. Low numbers of those who were referred take up the offer of a place in the intervention and complete it. Referral would usually be by a GP or practice nurse in a primary care setting. As patients with type 2 diabetes have an annual review appointment where behavioral change is meant to be discussed, it is likely that this would be the main setting for a weight management referral.

#### Step 2: Selecting the Target Behavior

This step was informed by work carried out locally in Glasgow by Rhonda Wilkie (MSc project, unpublished). Eleven patients who had not taken up the offer of a place in weight management were interviewed about their reasons for not doing so. A major theme was the initial discussion with a primary care health professional about weight and weight management (Textbox 2). Other issues that were raised by the patients such as service issues (eg, time and place of the intervention) and administrative issues (eg, not receiving invitations) were deemed outside the control of the working group and were the subject of other development work within the Health Board.

**Health professionals’ behaviors identified as potential targets and supporting quotes.**

**Not raising the issue of weight during a consultation or not doing so sensitively**

- “I avoid going to my GP [general practitioner] as the first thing I hear is [that] I should lose weight, which upsets me as my GP knows how much I’ve struggled...I have given up asking for help”

**Not informing patients they had been referred to weight management or what that involved (accurate information given)**

- “Ahh, she [GP] told me that I would be expected to attend for 2 years, every 2 weeks and eh, I wouldn’t be allowed to drop out, I had to guarantee that I would stay on the program for 2 years...she did tell me it was a class of 16 or 17 people all discussing it.” (incorrect information)
- “One bus, two trains and whatever transport I required from Queens park station to unit...if I had been referred nearer to home, (ie) Johnstone or Paisley, I certainly would have made the effort. Only I felt it ridiculous that a 71-year-old woman with health problems would be expected to travel to a different place to get to from her residence” (incorrect information)
- “Em, I think they [GP] just described Orlistat or whatever the drug is called and gave me a letter away with me. He didn’t go into what the service was or what you could do...

**Not supporting and encouraging patients to attend weight management and lose weight**

- “Lack of support from GP – I felt it was a way of dismissing my weight concerns, he delegated his responsibility by giving me a phone number...I have no support from my GP”

The target behavior selected was making “informed” referrals to weight management of patients with type 2 diabetes and high BMI (≥25 kg/m^2^) during annual diabetes review and supporting patients thereafter. It was felt that the impact of any behavior change is high, and there is a promising likelihood of being able to modify the behavior. There is also the additional benefit of spill-over to patients who have obesity but not type 2 diabetes and to other behavior change conversations such as smoking cessation. The ability to measure such a behavior change is high as the Glasgow and Clyde Weight Management Service uses electronic referrals meaning it is possible to see how many referrals are made, and “informed” can be inferred through uptake and attendance.

#### Step 3: Specify the Target Behavior

The target behavior, making “informed” referrals to weight management of patients with type 2 diabetes in primary care, needs to be performed by GPs and practice nurses, in the GP practice, during annual diabetes reviews, and during routine appointments if appropriate. The clinicians need to make more referrals to weight management, approach the subject sensitively and discuss it more often, encourage patients to take up referral and attend, and bring up the topic with patients more than once if required. They need to do this with every relevant patient (once a year per patient), working as a team between GP and nurse to decide who will do it.

#### Step 4: Identify What Needs to Change

This step was informed by qualitative interviews carried out as part of a health needs assessment by Public Health, NHS Greater Glasgow and Clyde, between October and December 2012. A total of 25 individuals were interviewed face to face and 2 by telephone. Participants included General Practice (Practice Nurses), Dietetics, Diabetes, Occupational Health, Cardiac Rehabilitation, Community Health (Health Improvement Team), Carers Service, Leisure Providers, and Rehabilitation. Table 1 outlines the components of the Capability, Opportunity, Motivation, and Behavior (COM-B) model with representative supporting quotations from the interviews where relevant.

**Table 1:** The components of the Capability, Opportunity, Motivation, and Behavior (COM-B) model, identified behaviors, and the need for change.

COM-B components	What needs to happen for the target behavior to occur?	Is there a need for change?	Supporting quote(s)
Physical capability	None	No change needed	N/A^a^
Psychological capability	Know local pathways and what else needs to be done prior to referral (eg, medication checks) To inform the patient of what is going to happen to them, referrers should know components of effective weight management	Yes, our qualitative research shows that referrers do not know local pathways or the components of effective weight management. N/A	“I don’t feel I’ve got the skills...it is such a specialist field...to lose weight is a big change. Sometimes the only enjoyment these people have in their life is actually food and to try and turn that on its head and see how they can support themselves, I don’t feel...I have the skills to do that effectively.” “I think further training would be better, even going to weight management classes to see what’s involved so I could then tell the patient...it’s alright me sitting with a patient and saying ‘you’ve got to lose weight’...just more about healthy eating, more in depth and if I was referring someone more information so I could then tell the patient, the more information the better.”
Physical opportunity	Have access to relevant patient materials to supplement referral discussions Have access to referral forms for weight management Have time to discuss the topic and make referral	Yes, currently such materials do not exist locally. No, referrals are done electronically via the standard referral system. Personal computers with this system are available in all consulting rooms in primary care and referrers will be used to using this system. Yes, make the discussion format as simple as possible to decrease the time required. Referral system is already quick and simple to use.	N/A N/A “Often patients I deal with have so many issues at one point in time, they couldn’t possibly think about dealing with weight management. I think that maybe means that we end up forgetting about it.”
Social opportunity	Make it part of the routine diabetes consultation carried out by all the practice team and the wider diabetes network Triggers to prompt discussion about weight and referral	Yes, would need to get everyone who sees patients with type 2 diabetes in the practice all discussing weight and making referrals. Ideally other practices (eg, within the quality improvement clusters) will also be doing this. No, there is already a prompt in the diabetes Chronic Disease Management Framework for behavior change, which includes recording discussions on weight management and referral to services.	N/A N/A
Reflective motivation	To want to discuss weight management and refer patients, feel that it is an essential part of their job, having confidence that they can discuss weight, and that it would be good for their patients	Yes, our qualitative research shows that there are issues with referrers’ motivation based on their beliefs towards weight management, their perceived role and belief in their abilities.	“I have no idea, that’s the truth…I’m limited in what I know. I can sit and discuss diet but priority in my role, I don’t know.”
Automatic motivation	Develop a habit of doing it	Yes, given referral rates are so low, it is clearly not yet a habit to discuss weight and refer patients during annual diabetes review.	N/A
Behavioral diagnosis of the relevant COM-B components	Psychological capability and reflective motivation are deemed to be the most important, with some changes required in physical opportunity, social opportunity and automatic motivation. Getting to the point where GPs and nurses want to discuss weight management and know how to discuss it and how to refer patients is key to addressing the problem	N/A	N/A

^a^N/A: not applicable.

#### Step 5: Identify Intervention Functions

The affordability, practicability, effectiveness/cost-effectiveness, acceptability, side-effects/safety, equity (APEASE) criteria were considered to select intervention functions. It was considered that education and training, and to a lesser degree environmental restructuring, would cover the range of behaviors identified in Step 4. Education was considered affordable, practical, and acceptable with a history of this intervention working in similar contexts. There are practical skills on the discussion of weight management that can be taught, and it is possible to develop patient materials that the referrers could have easily available.

#### Step 6: Identify Policy Categories

Using APEASE criteria, policy categories were considered for each of the selected intervention functions. Communication/ marketing and service provision (education), guidelines and service provision (training), and guidelines and environmental/ social planning (environmental restructuring) all fulfilled APEASE criteria.

#### Step 7: Identify Behavior Change Techniques

We identified five behavior change techniques matching the three intervention functions that were deemed necessary to improve the making of “informed” referrals to weight management of patients with type 2 diabetes in primary care: (1) information about health consequences, (2) prompts/cues, (3) demonstration of the behavior, (4) instruction on how to perform a behavior, and (5) adding objects to the environment.

#### Step 8: Mode of Delivery

Individual level and group face-to-face training and an individually accessed e-learning website were the only modes of delivery that met APEASE criteria. E-learning websites would allow the intervention to be delivered to many people and is a commonly used, acceptable mode of delivery in a health care setting. Face-to-face training allows for more in-depth skills training to be given.

**Figure 1:**
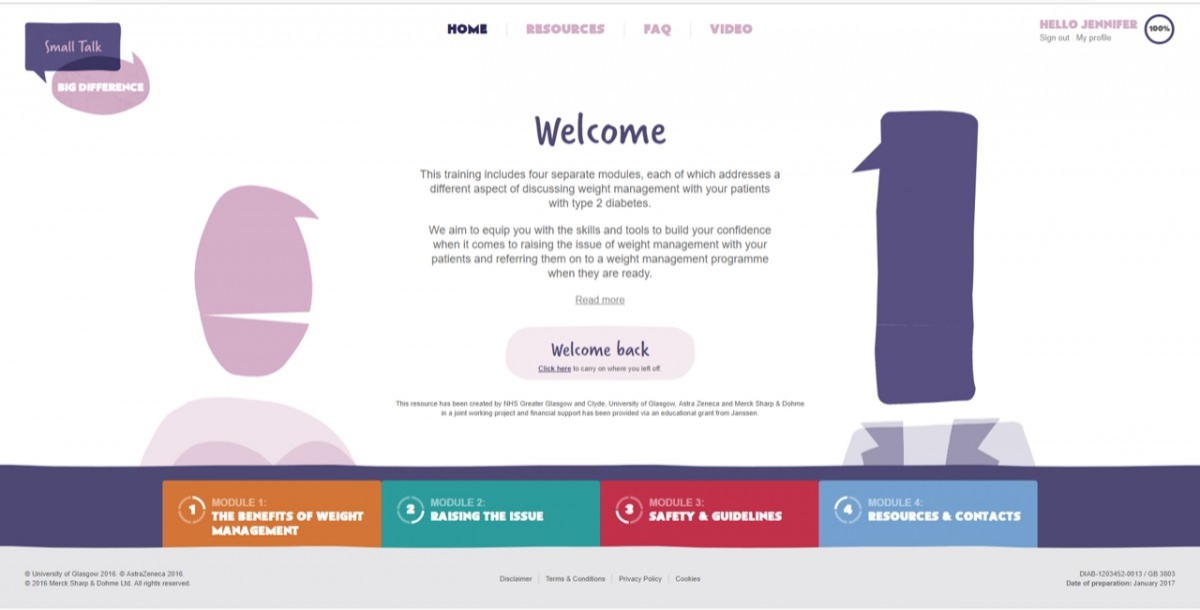
Welcome page from Small Talk Big Difference module showing the 4 available modules.

**Figure 2:**
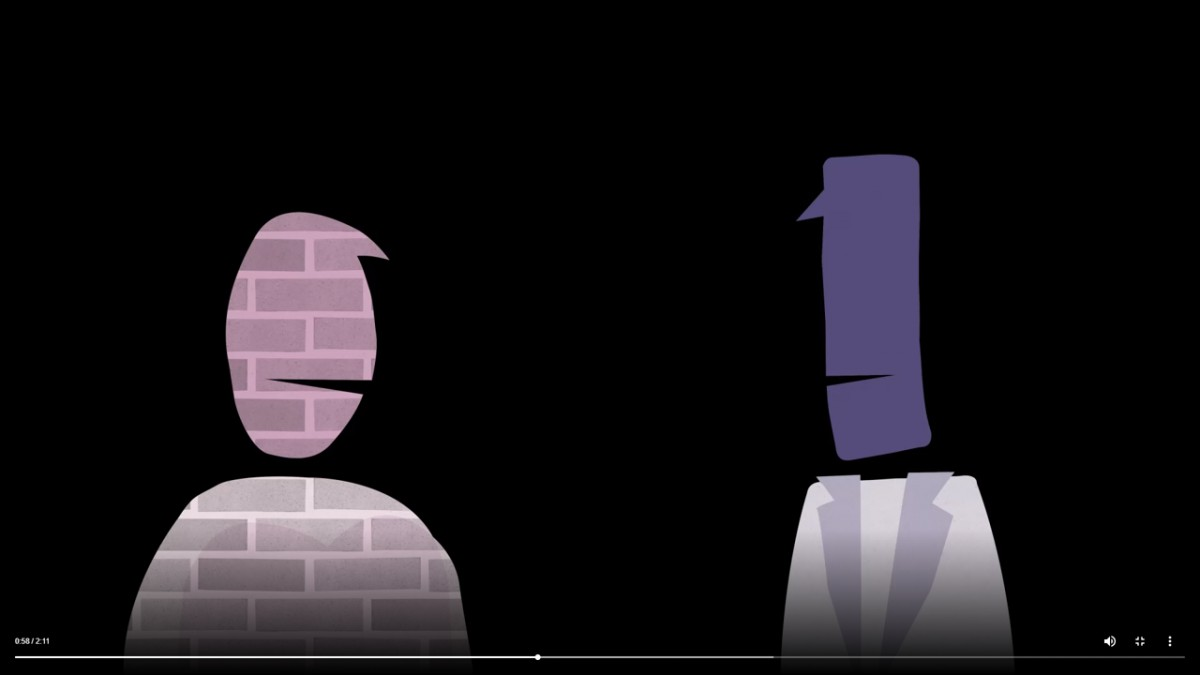
Screenshot from welcome video on Small Talk Big Difference eLearning platform.

**Figure 3:**
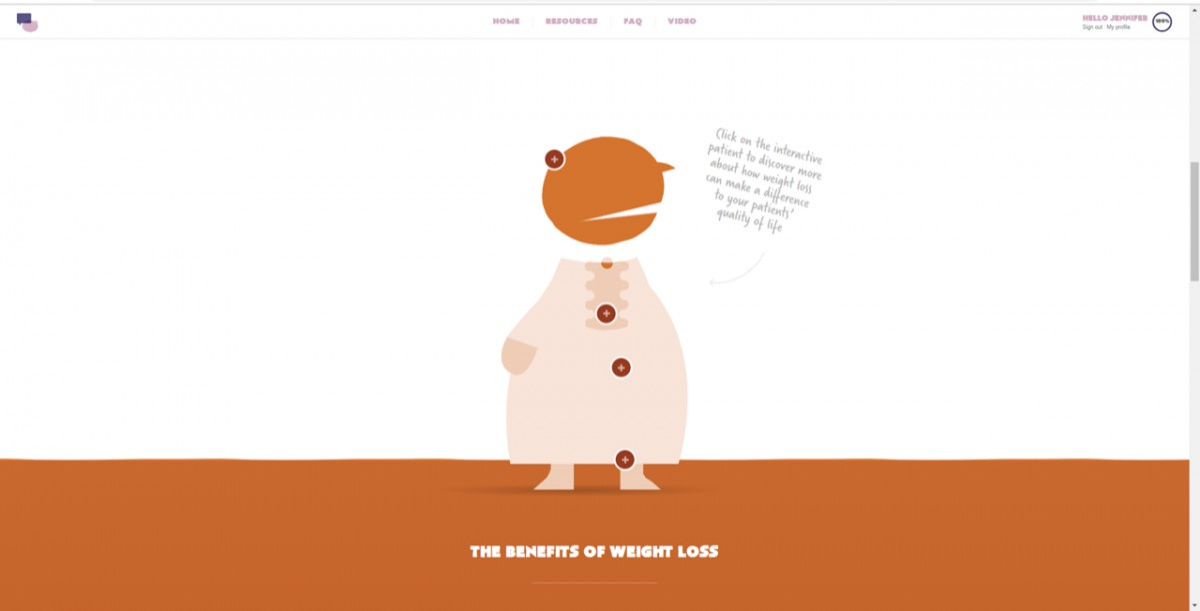
Screenshot from module on benefits of weight management in Small Talk Big Difference eLearning platform.

**Figure 4:**
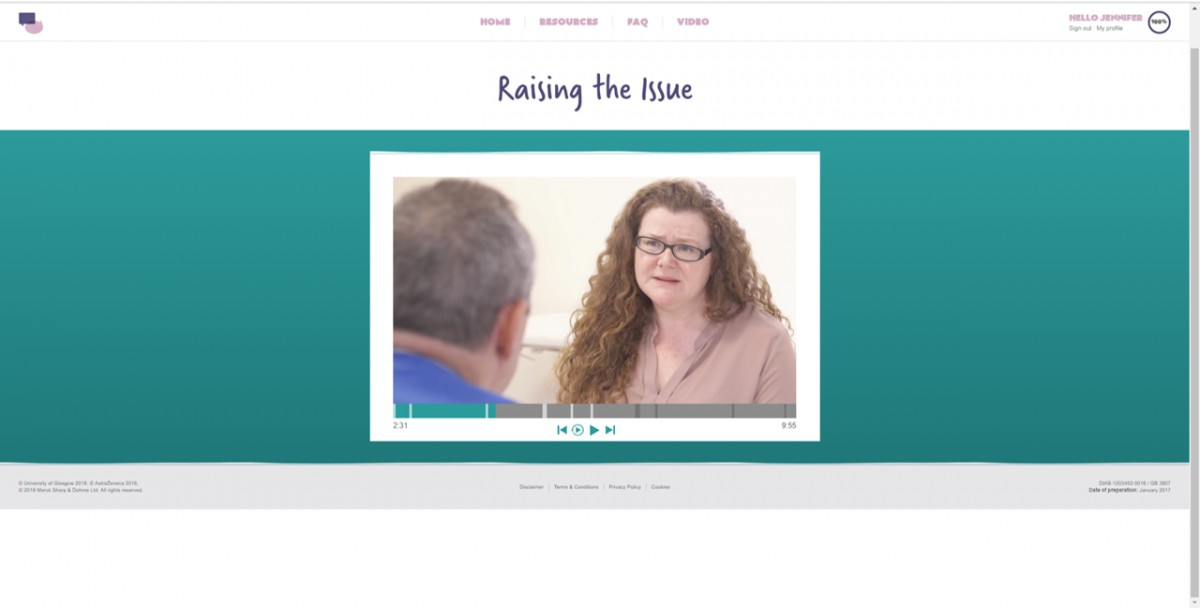
Screenshot from interactive video on communication skills in Small Talk Big Difference eLearning platform.

The Small Talk Big Difference (STBD) intervention comprises:

- a 1-hour Internet-based eLearning package covering the links between obesity, type 2 diabetes, and the benefits of weight management, the treatment of diabetes in patients with obesity, specific training in raising the issue of weight, local services and referral pathways, overview of weight management components/ evidence base, and the role of the referrer (Figures 1-4)
- training on raising the issue, with focus on three key components:
  1. Ask: seeking permission from the patient to discuss their weight
  2. Assess: determine if the patient thinks it important they manage their weight and how confident they feel about achieving weight loss; if the patient does not think it important or is not confident, provide further support and information and aim to discuss again in future
  3. Assist: make a referral to weight management and provide the patient with details of what will happen next and any requirements from the patient at this stage
- Internet-based training, which includes reference to recently published studies, case studies, and an interactive conversation with multiple choice questions to select the appropriate responses; learning will be assessed using end of module multiple choice questions
- a patient pamphlet covering the benefits of weight management in diabetes and what to expect during a weight management program
- a discussion tool with helpful facts and charts that can be used to guide a discussion about weight in a patient with type 2 diabetes
- a practice implementation checklist
- optional 2.5-hour face-to-face training building on the Internet-based module by using experiential learning to teach motivational interviewing techniques

### Trial Objectives

Our aims are to (1) evaluate the effectiveness of an Internet-based training program and practice implementation toolkit with or without face-to-face training for primary care staff in terms of patient attendance at NHS-funded adult weight management services, and (2) gain clinician feedback about an Internet-based training program, practice implementation toolkit, and face-to-face training on raising the issue of weight management with patients with type 2 diabetes.

### Trial Design

A randomized trial design will be used for this evaluation with GP practices randomly assigned to one of the two arms described. We will notify clinicians of the STBD package and evaluation through primary care management, training forums, and communications. If interested, the practices will be able to opt in to the STBD evaluation via email. Once a practice has expressed interest, they will then be randomized to one of the two arms and either receive immediate access to the Internet-based training and print materials or receive access 4 months later (with awareness that their referral rates will be monitored).

The inclusion criteria for participants requires that they be GP practices in NHS Greater Glasgow and Clyde that have a contract for local enhanced services for long-term conditions (ie, diabetes) and have a unique clinical database (ie, not shared with another practice). Practices classified as “17c” (those with a separate NHS contract for long-term conditions) and those practices with a database shared with another practice (8 practices in area) are excluded.

### Identification of Participants and Consent

As this is an evaluation study of a voluntary training program offered to GP practices as part of the usual program of NHS Health Improvement training, individual consent will not be required. By logging in to the Internet-based training website, practices will be agreeing to participate in the evaluation of the effectiveness of the training. No practices, staff, or patients will be identified during the evaluation. This information is in the letter instructing practices how to log into the training website. Patient data will be used to evaluate the outcomes of the training program, but this will be at the level of the intervention (ie, by GP practice) using health record linkage. The patient data and practice ID will be anonymized and accessed via the NHS Greater Glasgow and Clyde IT Safe Haven, so no individual patient consent will be sought.

All practices will be informed about the available training via communications from clinical directors and other routine NHS communication sources, which will explain the purpose of STBD and provide contact details to use if they are interested in completing the training. Those practices that do opt in will be randomized to one of the two arms of the evaluation. Practices gaining immediate access to the Internet-based training will be emailed with instructions on how to access the site (Arm 1). They will also be sent a practice kit containing the print materials designed to support implementation of STBD within their practice. Those practices randomized to Arm 1 will be sent options for the supplementary face-to-face training sessions and instructions on how to book a place through NHS Greater Glasgow and Clyde Health Improvement. Practices that express an interest and are randomized to Arm 2 (ie, delayed access to the STBD training), will be notified by email that their instructions for access will be available in 4 months. They will be made aware that their referral data over the next 4 months will be analyzed as part of the evaluation. A reminder letter/email/ phone call will be sent/made to any practice that opts in and does not access or complete the Internet-based training module after 4 weeks.

It will be made clear within correspondence with practices that completion of the training is entirely voluntary and that data for referrals, weight management, and diabetes outcomes will be examined to evaluate the impact of the new Internet-based training, but neither individual practitioners nor practices will be identified in any output.

### Trial Schedule

The schedule and timeline for the trial are outlined in Table 2.

**Table 2:** Trial schedule and timeline.

Activity	Owner	Outcome	Estimated timeline
Ad placed within primary care communication channels and forums	University of Glasgow and NHS^a^ Greater Glasgow and Clyde	Notify practice of training. Opt-in of practices to evaluation	Oct 2017
Randomization	University of Glasgow	Randomized practices that opt in to Arm 1 or 2 using Castor Electronic Data Capture	Oct 2017-Apr 2018
Practices notified of arm for evaluation	University of Glasgow	Practices informed if they are immediate or delayed access to training by email	Oct 2017-Apr 2018
Arm 1: Practices sent patient materials, posters, and invited to complete Internet-based training with or without face-to-face training	NHS Greater Glasgow and Clyde Health Improvement	Practices randomized to Arm 1 or 2 invited to complete training	Oct 2017-May 2018
Notification of completion of Internet-based training (Arm 1)	University of Glasgow	Website analytics notifies research team of Internet-based training completion by practice code	Oct 2017-Sept 2018
Reminder notice/phone call	University of Glasgow	Practices not having completed the Internet-based training will be sent a reminder and also the practice will be phoned once	Nov 2017-May 2018
Provision of face-to-face training (Arm 1)	NHS Greater Glasgow and Clyde Health Improvement	Notification of attendees provided to NHS team	A study-specific face-to-face training session available every 2 months Oct 2017-Jun 2018
Access to referral data	NHS Safe Haven	Statistician has remote access to anonymized data via a virtual private network	Oct 2018
Comparison of change in referral rate (Arms 1 vs 2)	Statistician	Report of results	Dec 2018

^a^NHS: National Health Service.

### Randomization

Practices will be randomized to immediate or delayed intervention using the Internet-based random allocation software (Castor Electronic Data Capture) using permuted random block sizes of 4.

### Outcome Measures

The primary outcome is change in primary care referral rate to an adult NHS weight management service. Using data from the weight management database held by NHS Greater Glasgow and Clyde, the ratio of referrals over 3 months before and after the STBD intervention (allowing 1 month for completion) will be calculated. Secondary outcome measures include change in referral, including uptake ratio, local enhanced service template completion (weight management discussed); change in local enhanced service template completion; completion of lifestyle weight management phase (completion defined as 80% attendance); weight change (kg and %) in lifestyle weight management phase for those attending >1 session; and weight change (kg and %) at 1 year for all patients (data from annual diabetes review). Tertiary outcomes include diabetes medications at time of referral, that is percentage on weight gaining medications (sulphonylureas, thiazolidinediones, insulin), and percentage on weight neutral/reducing medications (GLP-1 agonists, metformin, DPP-IV inhibitors, SGLT2 inhibitors). Exploratory analyses (before and after) will be carried out to look at the effect of completion of training; change in referral rate analysis of those who completed Internet-based training only; when training done was completed by GP only, by practice nurse only or both; and change in referral rate in those where one practice member completed Internet-based plus face-to-face training.

### Sample Size

A feasibility study was completed during which five practices (from a range of current referral rates to weight management services and deprivation areas in NHS Greater Glasgow and Clyde) were provided with access to the STBD e-learning program in June 2016. A GP and practice nurse from each attended a 2.5-hour face-to-face training session, and the practices were provided with the implementation toolkit. The referral rate to uptake was assessed for the 3 months prior to the STBD training (March-May 2016), 3 months post- STBD training (October-December 2016), and the same 3 months in 2015 (October-December 2015). We saw an increase in referrals of 50% compared to same period the year before (n=8 and 15 referrals respectively) and 88% compared to previous 3 months (n=10 and 15 referrals respectively) after the STBD intervention. Two practices did not make any referrals in the immediate time period analyzed prior to the intervention. As a result of this, together with the small number of feasibility practices, it was difficult to make a formal power calculation. The effect compared to same period 12 months before was 1.5 (SD 1.21); effect compared to 3 months previous was 1.88 (SD 0.38). A sample size of 80 per group would give 80% power to detect a difference of 0.5 in the change in referral rate based on a hypothetical SD of 1.12.

### Statistics

Comparisons between study arms will be by independent two-sample *t* tests or appropriate nonparametric equivalents using the prespecified comparisons.

### Study Closure

The evaluation will end 15 months after the practices are invited to participate, to allow time for training, changes in practice, and patient attendance at services.

### Data Handling

We have prepared a custom electronic case report form for the purposes of randomizing the practices to immediate or delayed access to STBD training at the point of opting in. The remaining data for use in this evaluation will be from routine health records (ie, weight management and diabetes care). Access to these data will be via the NHS Greater Glasgow and Clyde Safe Haven (already approved by the Local Privacy Advisory Committee). The data will be fully anonymized and accessed via a virtual private network. Access will be only for the duration of the analysis of the data for the evaluation.

### Review of the E-Learning, Patient Materials, and Evaluation

The STBD training and patient materials packages were developed over 2 years. The contents of the materials were developed by the NHS clinical team including a consultant physician, a GP, a consultant psychologist, specialist dietitian, and a health improvement specialist. Review has been extensive with patient materials reviewed by 7 patient volunteers (with type 2 diabetes and co-existing obesity), the training materials were reviewed by 3 specialist dietitians and 3 health improvement specialists, and then by 8 GPs who are based outside of NHS Greater Glasgow and Clyde. All materials were extensively reviewed by medical and marketing staff from AstraZeneca and Merck Sharp & Dohme to ensure they complied with the Association of British Pharmaceutical Industry code of practice. Evaluation plans had oversight from the evaluation team within NHS Greater Glasgow and Clyde Public Health. After the pilot study was conducted, the e-learning was independently reviewed and approved for continuous professional development points from both the Royal College of Nursing and the Royal College of General Practitioners.

### Approvals

Favorable ethical opinion was received from London – Bromley Research Ethics Committee. Sponsorship and management approval were received from NHS Greater Glasgow and Clyde. NHS Greater Glasgow and Clyde and Glasgow University Insurance and Indemnity will apply.

## Results

The first invitations for this evaluation were emailed in October 2017. Recruitment for this trial closed in April 2018. As there was an extremely large response to the single invitation email from the Primary Care Diabetes Lead sent in March 2018, to avoid discouraging willing practices we decided to extend the trial to include 100 practices (50 per arm). Follow-up is ongoing and linkage of practice data with referral data was completed by October 2018. It is expected that the trial will report in early 2019.

## Discussion

### Principal Considerations

While the concept of providing training in behavior change techniques and of promoting referrals to services such as weight management to staff working in primary care is not new, to date there have been no interventions that have been evaluated to assess if they are effective at improving rates of referrals and clinical outcomes for patients [17]. While the primary outcomes will be change in referral rates to weight management, secondary outcomes will include weight change and diabetes medications outcomes for referred patients, obtained via health record linkage and de-anonymized. This trial comes at a time when there is strong interest both in weight management for people with type 2 diabetes and in reducing weight stigma in health care interactions and more widely. New interventions for weight management and type 2 diabetes show very promising results [9,18,19]; however, they are Phase 2 and 3 trials with recruited volunteers. The challenge occurs when moving these interventions to real-world settings. To access the benefits of such new interventions, the patients’ usual care providers, normally primary care, will have to raise the issue and discuss weight, weight management, and know how and where to refer patients to. Unless this becomes universal, there will be inequity of access to these promising new treatments.

The trial is an evaluation of an intervention that has been developed and delivered by the health care teams usually responsible for delivering practitioner education. All email communications inviting practices to participate were sent by the usual primary care communication channels in the usual weekly newsletters. There was no separate consent process and no research visits or assessments. The entire process has been true to how it would be delivered outside of the intervention. By including 100 of 260 practices in the area, we believe it will be representative of primary care, rather than just an interested few practices.

### Limitations

One current limitation is the lack of a detailed process evaluation. We have some useful data available such as details of who has accessed the website and completed each of the modules, records of any requests for additional patient pamphlets or other materials, and full details of referral, uptake, and completion of weight management alongside electronic diabetes care records. If the intervention is not shown to be effective, it will be important to better understand what contributed to that lack of success, probably requiring interviews with practitioners and patients and even observation or recording of consultations.

### Conclusion

We hope that if this evaluation shows that the Small Talk Big Difference intervention is effective, it will be shared with other health bodies across the United Kingdom and beyond for wide dissemination. It is locally editable and easy to update, meaning that it could be modified to fit other health systems if required.

## Abbreviations

APEASE: affordability, practicability, effectiveness/cost-effectiveness, acceptability, side-effects/safety, equity
BMI: body mass index
COM-B: Capability, Opportunity, Motivation, and Behavior
GP: general practitioner
NHS: National Health Service
STBD: Small Talk Big Difference
